# Characterization of pKPN945B, a novel transferable IncR plasmid from hypervirulent carbapenem-resistant *Klebsiella pneumoniae*, harboring *bla*_IMP-4_ and *qnrS1*

**DOI:** 10.1128/spectrum.00491-24

**Published:** 2024-09-17

**Authors:** Xue Xiao, Chunlin Feng, Jingchen Hao, Ling Cheng, Chunxia Jian, Zhangrui Zeng, Jinbo Liu

**Affiliations:** 1 Department of Laboratory Medicine, The Affiliated Hospital of Southwest Medical University, Luzhou, China; 2 Sichuan Province Engineering Technology Research Center of Molecular Diagnosis of Clinical Diseases, Luzhou, China; 3 Molecular Diagnosis of Clinical Diseases Key Laboratory of Luzhou, Luzhou, China; 4 Hospital-Acquired Infection Control Department, Affiliated Hospital of Southwest Medical University, Luzhou, China; Central Texas Veterans Health Care System, Temple, Texas, USA

**Keywords:** hypervirulent carbapenem-resistant *Klebsiella pneumoniae*, *bla*_IMP-4_, IncR, transferable

## Abstract

Carbapenem-resistant *Klebsiella pneumoniae* producing metallo-β-lactamase poses a major public health threat worldwide. Imipenemase often coexists with other resistance genes leading to the formation of multidrug-resistant bacteria. In this study, we describe the microbiological and genomic characteristics of the hypervirulent carbapenem-resistant *K. pneumoniae* ST20-K23 strain KPN945 harboring *bla*_IMP-4_ and *qnrS1*. The minimum inhibitory concentration of KPN945 against antimicrobials was determined by the broth microdilution method. The virulence of KPN945 was evaluated through string test, serum killing resistance, and *Galleria mellonella* larvae infection models. The transferability of pKPN945B was assessed using a conjugation test. The genome sequence characteristics of KPN945 were analyzed through whole genome sequencing, and a phylogenetic tree was constructed to evaluate the prevalence of imipenemase. Our findings showed that KPN945 was non-susceptible to β-lactam antibiotics, highly resistant to serum killing, and highly lethal to *G. mellonella* larvae. The fusion plasmid pKPN945B carried by the isolate KPN945 belonged to the IncR incompatibility group and harbored multiple drug resistance genes such as *bla*_IMP-4_, *bla*_CTX-M-14_, *qnrS1*, and *sul2*. The most important point is that the IncR plasmid is a novel plasmid that arose by the accretion of parts from different plasmids, making it transferable and with a fitness cost. Globally, *bla*_IMP-4_ is the most prevalent imipenemase subtype, with the highest isolation rates in Asia, particularly China. The spread of *bla*_IMP-4_, especially the emergence of transferable plasmids, deserves our vigilance and prevention. Additionally, we should pay attention to the formation of hypervirulent *K. pneumoniae* mediated by non-virulent plasmids.

**IMPORTANCE:**

Up to now, IncR replicons carrying *bla*_IMP-4_ have not been reported, and the IncR plasmids described in previous studies have been found to be non-transferrable to other bacteria through conjugation. Moreover, there have been no extensive phylogenetic analyses of strains carrying blaIMP in the published papers. The lack of data in these studies is noteworthy because blaIMP appears in the novel transferable fusion plasmid IncR. Although the IncR plasmid has no tra operon, it can still be transferred to *Escherichia coli* EC600 or *Klebsiella* pneumoniae ATCC13883 (RIF^R^) without high fitness cost, but it only affects the MIC of imipenem. *bla*_IMP_ integrates with other resistance mechanisms leading to the formation of multidrug-resistant strains. Notably, the high prevalence of *bla*_IMP-4_ in Asia and the presence of *bla*_IMP-4_ on novel transferable IncR plasmids suggest the urgent need to monitor the emergence of such plasmids and control their spread.

## INTRODUCTION

*Klebsiella pneumoniae* is one of the most common pathogens of nosocomial infection, often leading to pneumonia, urinary tract infection, and liver abscess (1). The emergence of carbapenem-resistant *K. pneumoniae* (CRKP) has reduced clinical treatment options and increased patient mortality. One of the resistance mechanisms of CRKP is the production of metallo-β-lactamase (MBLs), of which the most common MBL in China is New Delhi MBL (NDM) (2), and few imipenemase MBLs (IMP) cause outbreaks of infection. Besides, IMP-harboring CRKP leads to low levels of carbapenem resistance (3, 4), making it easily overlooked, thereby underestimating the rate of IMP presence in *K. pneumoniae. K. pneumoniae* is not the most common host of IMP in China. The first reported IMP-type enzyme in China, *bla*_IMP-4_, was found in *Citrobacter youngae* (5). IMP is more common in *Pseudomonas aeruginosa*, as reported in South America (6), the United States (7), Canada (8), Australia, and the Czech Republic (9), followed by *Acinetobacter baumannii* and *K. pneumoniae* (10).

*K. pneumoniae* carrying IMP-type enzymes are often associated with some rare sequence types, such as ST476, ST1114, ST686, and ST323 (11). There are currently 102 variants of the IMP enzyme (available online:http://www.bldb.eu/, accessed on 8 November 2023), with IMP-1 being the most common variant. Most of the *bla*_IMP_ genes identified came from hospital isolates, with the highest prevalence in Japan, followed by China. In China, the most common variant is IMP-4 (10). The *bla*_IMP_ genes are typically found in class 1 integrons carried by plasmids (IncN-type plasmids), and these integrons play a crucial role in the maintenance and spread of resistance in Gram-negative bacteria (12). Moreover, most IMP gene cassettes are located near the Pc promoter of the class 1 integrons, facilitating their expression in different hosts (12). While most studies report that IMP generally leads to low levels of carbapenem resistance, it often works in conjunction with other resistance mechanisms to promote the development of multidrug resistance phenotypes (4). The combination of resistance and hypervirulence phenotypes found in IMP-producing CRKPs, as well as the inherent resistance of MBLs (including IMP) to ceftazidime/avibactam (CZA) (a novel β-lactamase inhibitor), will further emphasize the importance of monitoring the emergence of such bacteria.

IncR replicons generally have conserved skeletons, with regions responsible for plasmid replication, maintenance, and stabilization, including *rep*B (replicating protein), *parAB* (involved in the partitioning and compatibility of the plasmid), *umuCD*, and *vagCD* (toxin-antitoxin system) (13, 14). However, IncR replicons generally lack conjugation transfer systems. They cannot be transferred through conjugation, but they can form fusion plasmids with other incompatible plasmids such as IncFII or IncN3, which is conducive to transmitting the harbored resistance genes (15–17). To date, IncR replicons have been found to harbor a variety of drug resistance genes, such as *bla*_VIM-11_ (18), *bla*_KPC-2_ (19), *qnr*, *bla*_TEM-1_, and *rmtB*. Notably, IncR plasmids carrying *bla*_IMP-4_ have not been reported.

The isolate KPN945, isolated from a southwest teaching hospital, harbored *bla*_IMP-4_ and *qnrS1*, which were located on an IncR-type plasmid. As far as we know, no IncR plasmid carrying *bla*_IMP-4_ has been reported. KPN945 was initially identified using Matrix-Assisted Laser Desorption/Ionization Time of Flight Mass Spectrometry (MALDI-TOF-MS), and further microbiological characterization and whole genome sequencing were conducted to assess its characteristics and understand the mechanism of transmission.

## MATERIALS AND METHODS

### Bacterial isolates and antimicrobial susceptibility testing

A CRKP isolate, designated as KPN945, was collected from a teaching hospital in Southwest China. The isolates were identified using MALDI-TOF-MS (German, Bruker). The broth microdilution method was used to determine the MIC of ceftazidime (CAZ), meropenem (MEM), ertapenem (ETP), imipenem (IPM), aztreonam (ATM), CZA, tetracycline (TET), penicillin (PEN), ampicillin (AMP), piperacillin (PIP), cefazolin (CFZ), cefuroxime (CXM), gentamicin (GEN), levofloxacin (LVX), chloramphenicol (CHL), polymyxin B (PMB), and tigecycline (TGC). The standard strain *Escherichia coli* ATCC25922 was used as the control strain. The MIC of the isolates was interpreted using the drug-susceptibility breakpoints recommended by the Clinical and Laboratory Standards Institute (20). The TGC MIC was interpreted using the Food and Drug Administration breakpoint.

Efflux pump inhibition tests were conducted on drug-resistant strains to assess the contribution of the efflux pump to drug resistance. The efflux pump inhibitor was 1-(1-naphthyl methyl)-piperazine (NMP, 100 mg/L) (China, Alfa Aesar). If the MIC of an antimicrobial combined with NMP was reduced by fourfold or more compared with that of the antimicrobial alone, it indicated efflux pump works.

### Screen carbapenemase and DNA amplification

The modified carbapenemase inactivation method (mCIM) and EDTA-modified carbapenemase inactivation method (eCIM) were employed according to the (20) standards. Simultaneous detection of eCIM and mCIM can differentiate between MBLs and serine carbapenemase in *Enterobacteriales*. Briefly, isolate KPN945 was inoculated into 2 mL of Trypticase Soy Broth (TSB) and 2 mL of TSB supplemented with 20 µL of 0.5 M EDTA. A 10-µg MEM disk was immersed into the two test tubes and incubated for 4 h at 37°C. The 0.5 McFarland (McF) *E. coli* ATCC25922 was coated on the Mueller-Hinton Agar (MHA) plate. After drying, the MEM disk was picked out and squeezed out water and then pasted on the MHA plate. The plate was then incubated at 37°C for 18–24 h, after which the size of the inhibition zone was measured. A difference in the inhibition zone of ≥5 mm between the eCIM and mCIM tests indicated positive MBL production by KPN945, while a difference of ≤4 mm indicated no MBL production.

The DNA of isolate KPN945 was extracted by boiling pyrolysis. The general procedure is to pick a loop of bacteria and add 500 µL of sterile water and mix, centrifuge at 12,000 *g* for 5 min, remove the supernatant, then add 500 µL of sterile water again, vortex and mix, boil at 100°C for 10 min, centrifuge at 12,000 *g* for 10 min, and collect the supernatant as DNA template. PCR and agarose gel electrophoresis were used to detect the presence of *bla*_KPC_, *bla*_NDM_, *bla*_IMP_, *bla*_VIM_, or *bla*_OXA-48_ in isolate KPN945. Primer sequences for amplification are shown in Table S1.

### String test and serum killing assay

The hypermucoid phenotype of isolate KPN945 was assessed using the string test. A single purified colony was picked from a blood plate with an inoculation loop. If the length of the viscous string of the colony was longer than 5 mm, the string test was considered positive (21).

The *in vitro* virulence of KPN945 was evaluated through a serum killing test. 0.5 McF bacterial solution was diluted 100-fold in Luria-Bertani (LB) broth. This solution was then mixed with healthy human serum at a 1:3 ratio and incubated for 0 h, 1 h, 2 h, and 3 h. The plates were inoculated with the mixture, and the number of visible colonies was counted. According to the count results, the isolates can be classified as “highly sensitive,” “moderately sensitive,” or “highly resistant” to serum, based on the criteria of previous studies (22).

### *Galleria mellonella* larvae infection model

As previously described (23), the pathogenicity of KPN945 was assessed by the *G. mellonella* larval infection mode. Fifteen healthy vigorous larvae were inoculated with the bacterial suspension at a concentration equivalent to 10^5^ CFU/mL and observed for 3 days. When the larvae were inactive and black, they were considered dead. The number of larval deaths was recorded every 12 h. Survival curves were generated using GraphPad Prism9.

### Conjugation experiments

The horizontal transfer ability of the pKPN945B plasmid carrying *bla*_IMP-4_ gene was evaluated by conjugation experiments. Rifampicin (RIF)-resistant *E. coli* EC600 or TGC-resistant *Pseudomonas aeruginosa* PAO1 were used as recipients, while KPN945 served as the donor for conjugation experiments. The ratio of LB broth, donor bacteria, and recipient bacteria was 4:2:1. The mixture was then incubated at 37°C for 16–20 h. Subsequently, 100 µL of bacterial mixture was evenly coated on LB agar medium (500 mg/L RIF, 500 mg/L RIF + 1 mg/L MEM; 4 mg/L TGC, and 4 mg/L TGC + 2 mg/L IPM) for overnight culture. Colonies grown on 500 mg/L RIF + 1 mg/L MEM or 4 mg/L TGC + 2 mg/L IPM medium were randomly selected and identified by MALDI-TOF-MS, colony PCR, and Sanger sequencing to confirm the success of conjugation. For the colony PCR reaction system, 2× PCR SuperMix 12.5 µL, ddH_2_O 10.5 µL, *bla*_IMP-4_ F + R primers 2 µL, and the colonies were dipped into reaction system and mixed. Additionally, we extracted the plasmid from the transconjugants following the manufacturer’s protocol (Omega, Norcross, USA). The plasmid DNA served as the template for PCR, and agarose gel electrophoresis was conducted to verify the presence of *bla*_IMP-4_ on the plasmid. If it was present, it further indicated that the conjugation was successful.

The *K. pneumoniae* standard strain, ATCC13883, was continuous passage cultured in LB broth containing a series of concentration gradients of RIF to induce RIF resistance. The induced ATCC13883 (RIF^R^) was used as the recipient and KPN945 as the donor bacterium, and the conjugation experiments were performed again, as described above.

### Growth kinetics assay

The growth kinetics of the ATCC13883 (RIF^R^) and transconjugants (ATCC13883-pKPN945B) were assessed to determine the fitness cost of acquiring a resistant plasmid. Simply put, 10 µL of 0.5 McF bacterial suspension was mixed with 190 µL of LB broth, incubated at 37°C, and optical density at 600 nm (OD_600_) was measured every hour for a total of 12 h.

### Whole genome sequencing

The genomic DNA of KPN945 was extracted using the Wizard Genomic DNA Purification Kit (Promega) according to the manufacturer’s protocol. Purified genomic DNA was quantified by TBS-380 fluorometer (Turner BioSystems Inc., Sunnyvale, CA). The genomic DNA of isolate KPN945 was sequenced using a combination of PacBio Sequel II and Illumina sequencing platforms. The prepared libraries then were used for paired-end Illumina sequencing (2 × 150 bp) on an Illumina Novaseq6000 machine. For PacBio sequencing, an aliquot of 10 µg DNA was spun in Covaris G-tubes (Covaris, MA) at 6,000 rpm for 60 s using an Eppendorf 5424 centrifuge (Eppendorf, NY). DNA fragments were then purified, end repaired, and ligated with SMRT bell sequencing adapters following the manufacturer’s recommendations (Pacific Biosciences, CA). The resulting sequencing library was purified three times using 0.45× volumes of Agencourt AMPure XP beads (Beckman Coulter Genomics, MA) following the manufacturer’s recommendations. Next, a ~10-kb insert library was prepared and sequenced on one SMRT cell using standard methods.

The raw Illumina sequencing reads generated from the paired-end library were subjected to quality filtering using fastp v0.23.0. The raw sequencing reads generated from the PacBio platform were processed using SMRT Analysis v2.3.0. Then, the clean short and long reads were coassembled to construct complete genomes using Unicycle v0.4.8 (24). The coding sequences (CDs) of chromosome and plasmid were predicted using Prodigal v2.6.3 (25) and GeneMarkS (26), respectively. tRNA-scan-SE (v 2.0) (27) was used for tRNA prediction, and Barrnap v0.9 was used for rRNA prediction. The predicted CDs were annotated from NR, Swiss-Prot, Pfam, GO, COG, and KEGG databases using sequence alignment tools such as BLAST, Diamond, and HMMER.

The antimicrobial resistance genes, virulence genes, plasmid replicon typing, and Multi-locus sequence typing (MLST) of the isolates were analyzed on http://genomicepidemiology.org/, https://bigsdb.pasteur.fr/, and http://www.mgc.ac.cn/cgi-bin/VFs/v5/main.cgi. ISfinder (https://www-is.biotoul.fr/) was used to analyze the insertion sequence. https://blast.ncbi.nlm.nih.gov/Blast.cgi, BRIG, Easyfig, and Snapgene were used for genome comparison. *K. pneumoniae* ATCC13883 and *Klebsiella quasipneumoniae* ATCC700721 were used as standard strains to compare point mutations of isolates. The Species Tree Inference from All Genes (28) was adopted to construct a phylogenetic tree based on the core genome, and the OrthoFinder (29) was used for phylogenetic tree analysis. The specific information of the strains (IMP type, accession number, taxonomy, country, and source) is shown in Table S3. The sequencing results were posted on https://www.ncbi.nlm.nih.gov/.

### Quantitative reverse transcription-PCR

In brief, single-purified colonies of the target strain were inoculated into 5 mL of LB broth and grown to the log phase. RNA extraction was carried out according to the manufacturer’s instructions (Magen, China). Then, the isolated total RNA was reverse transcribed using a High-Capacity cDNA Reverse Transcription Kit. The quantitative reverse transcription-PCR (qRT-PCR) primers used in this study are listed in Table S1. The *rpoB* gene served as an internal reference. Gene expression results were calculated using the −2 ^ΔΔCt^ method and analyzed using GraphPad Prism 9.

## RESULTS AND DISCUSSION

### Antimicrobial susceptibility and general characteristics of KPN945

**Fig 1:**
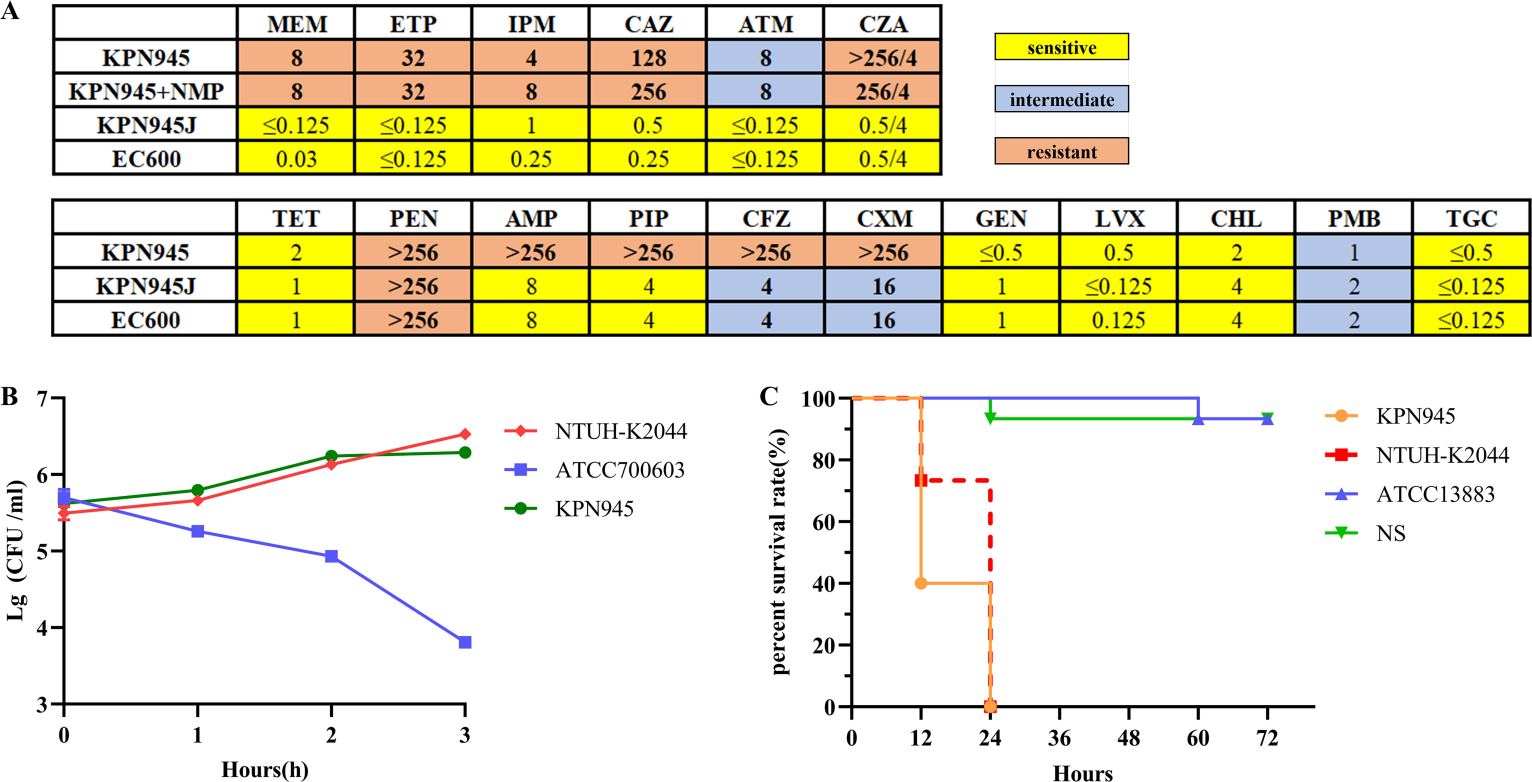
Results of antimicrobial susceptibility test, serum killing resistance, and pathogenicity of KPN945. (**A**) Antimicrobial susceptibility tests and (or) efflux pump phenotype test of KPN945, transconjugants (KPN945J), and EC600. The number represents the MIC (mg/L). (**B**) Three-hour survival rate of NTUH-K2044, ATCC700603, and KPN945 in healthy human serum. NTUH-K2044 was the positive control, and ATCC700603 was the negative control. (**C**) The survival rate of *G. mellonella* larvae (15 biological replicates) inoculated with KPN945, NTUH-K2044, ATCC13883, and normal saline (NS).

Isolate KPN945 was resistant to β-lactam antibiotics, including carbapenems, while remaining susceptible to aminoglycosides (GEN), fluoroquinolones (LVX), CHL, TET, PMB, and TGC (Fig. 1A). When treated with the efflux pump inhibitor NMP, the MIC changes of KPN945 on CAZ, MEM, ETP, IPM, ATM, and CZA did not exceed four times or more, indicating that the efflux pump did not significantly contribute to these drug resistances.

The difference in inhibition zone between mCIM and eCIM was >5 mm, suggesting that isolate KPN945 carried MBLs (Table S2). Subsequently, PCR and Sanger sequencing confirmed the presence of *bla*_IMP-4_ in KPN945.

Isolate KPN945, belonging to the K23-ST20 lineage, exhibited non-mucoid colonies on blood agar plates. Although it was negative for the string test (the length of viscous strings < 5 mm), it was highly resistant to serum killing (Fig. 1B) and highly lethal in the *G. mellonella* larva infection model (Fig. 1C). This indicated that isolate KPN945 was a hypervirulent *K. pneumoniae*. Table 1 summarizes the above characteristics.

**TABLE 1:** Microbiological characteristics from the isolate KPN945

Characteristics	KPN945
Sequence type	ST20
Serotype	K23
Carbapenemase gene	*bla*_IMP-4_
Other resistance determinants	
Beta-lactam	*bla*_CTX-M-14_, *bla*_LAP-2_, *bla*_SHV-187_
Sulfonamides	*sul2*
Fluoroquinolones	*qnrS1*
Aminoglycosides	*aph (6)-Id*, *aph (3”)-Ib*
TET	*/^a^*
Others	*fosA*
Plasmid Inc types	IncHI1B; IncFIB(K); IncR; Col(pHAD28)
Virulence genes	*iutA*, *mrk*, *fim*, *ent*, *fep*, *fes*, *iroEN*, T6SS, LPS rfb locus, etc.
String test	Negative
Serum killing	Highly resistant
Mortality rate of *G. mellonella*	100%

^*a*^ /- not detected.

### Antimicrobial susceptibility changes and fitness characteristics of transconjugants

We conjugate the drug-resistant plasmid into *E. coli* EC600, and the verification of the conjugation results is shown in Fig. S1. We selected colonies that grew on the 500 mg/L RIF + 1 mg/L MEM plate. Since KPN945 was susceptible to RIF but resistant to MEM and the recipient bacterium EC600 was resistant to RIF and susceptible to MEM, neither KPN945 nor EC600 would grow on the plate when they existed alone. Only when EC600 successfully obtained the resistant plasmid pKPN945B from KPN945 did the corresponding colony grow on the plate. The colonies were then selected and identified by MALDI-TOF-MS, which confirmed they were *E. coli*. PCR and Sanger sequencing revealed that EC600 harbored *bla*_IMP-4_, a drug-resistant gene present on plasmid pKPN945B, indicating successful conjugation. Furthermore, the plasmid DNA of the transconjugants also carried *bla*_IMP-4_, further emphasizing successful conjugation. The conjugation screening of ATCC13883 (RIF^R^) as recipient bacteria was the same as above, and the screening results are shown in Fig. S1. The pKPN945B can be transferred to *E. coli* EC600 at a transfer frequency of about 6.34 × 10^−5^. We found that the presence of *bla*_IMP-4_ resulted in decreased susceptibility to IPM (0.25 mg/L to 1 mg/L) and had no significant effect on the susceptibility of other antibiotics (Fig. 1A). In addition, the sensitivity of transconjugants ATCC13883-pKPN945B to IPM was also reduced compared with ATCC13883 (RIF^R^) (0.5 mg/L to 1 mg/L). The presence of *bla*_IMP-4_ in KPN945 led to a low level of carbapenem resistance, and in the transconjugants, it only resulted in decreased susceptibility to IPM, indicating that other factors combined with *bla*_IMP-4_ led to β-lactam resistance in KPN945. Compared with ATCC13883 (RIF^R^), the transconjugants ATCC13883-pKPN945B showed a fitness cost (Fig. S2). Studies have shown that the fitness cost of carrying plasmids is related to the strength of selection for plasmid-encoded traits, such as antibiotic resistance or virulence (30). The plasmid pKPN945B only caused a four- (or two-) fold increase in the MIC level of IPM; this may be the reason why the transconjugants carry pKPN945B without high fitness cost. It is necessary to control the spread of *bla*_IMP-4_ and prevent its integration with other resistance mechanisms within the same cell.

### Genomic feature of the isolate KPN945

The total length of the KPN945 genome is 5,511,741 bp, consisting of a chromosome backbone (5,205,392 bp) harboring *bla*_SHV-187_ and three circular plasmids (Table 2). The plasmid incompatibility (Inc) groups of plasmids pKPN945A (225,147 bp), pKPN945B (78,707 bp), and pKPN945C (2,495 bp) were HI1B/FIB(K), R, and Col(pHAD28), respectively. The *bla*_IMP-4_ gene is located on pKPN945B. In addition, genes mediating resistance to cephalosporins (*bla*_CTX-M-14_), PEN (*bla*_LAP-2_), fluoroquinolones (*qnrS1*), sulfonamides (*sul2*), and aminoglycosides [*aph (6)-Id*, *aph (3 ″)-Ib*)] were also found in pKPN945B. The virulence genes *iutA* and *mrkABCDFHIJ* were located on the chromosome backbone of KPN945, and no virulence factors such as *rmpA*/*A2* and *iucABCD* were detected.

**TABLE 2:** Genomic feature of the isolate KPN945^*a*^

ID	Length (bp)	G + C (%)	Resistance genes	Incompatibility group	Sequence shape
Chromosome	5,205,392	57.62	*bla*_SHV-187,_ *fosA*	NA^*a*^	Circle
pKPN945A	225,147	51.04	/^*b*^	IncHI1B/ IncFIB(K)	Circle
pKPN945B	78,707	50.19	*bla*_IMP-4_, *bla*_CTX-M-14_, *bla*_LAP-2_, *qnrS1*, *sul2*, *aph (6)-Id*, *aph (3'')-Ib*	IncR	Circle
pKPN945C	2,495	51.46	/^*b*^	Col(pHAD28)	circle

^*a*^ NA, not applicable.

^*b*^ /- not detected.

Although pKPN945B carried *qnrS1*, the isolate KPN945 remained susceptible to fluoroquinolone (LVX). *qnrS1* harbored by pKPN945B showed 100% identity and coverage with *qnrS1* harbored by other strains in the National Center for Biotechnology Information (NCBI) database, and *gyrA* (I872V, E874D) and *parC* were found without known mutations compared with ATCC13883. Some studies have reported that the combination of *qnrS1* with efflux pump *oqxAB* can cause non-susceptibility to fluoroquinolones (31), but the qRT-PCR results in this study revealed decreased expression of *oqxAB* in the isolate KPN945 (Fig. S3), suggesting that *qnrS1* alone may still maintain susceptibility to fluoroquinolones (LVX). This is consistent with previous research (31, 32).

According to ResFinder 4.1, KPN945 was found to have mutations in OmpK36 and OmpK37, and OmpK36 (A217S) and OmpK37 (I70M, I128M) mediated carbapenem resistance, indicating that *bla*_IMP-4_ and outer membrane porins collectively contribute to carbapenem resistance.

### Sequence analysis of plasmid carried by isolate KPN945

**Fig 2:**
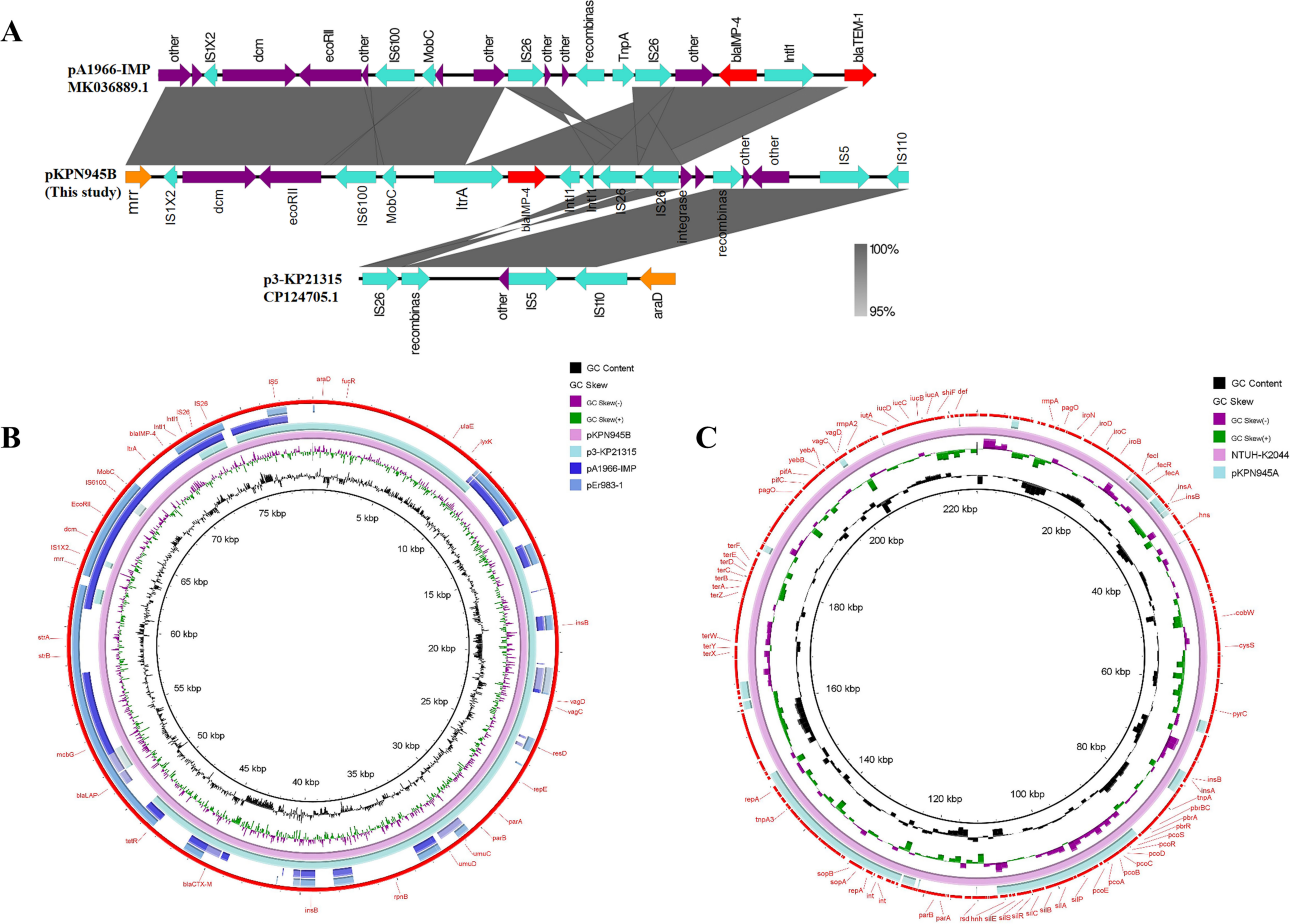
Genetic environment of *bla*_IMP-4_. Circular comparison of plasmids pKPN945B and pKPN945A. (**A**) Comparison of genetic elements around *bla*_IMP-4_ between pKPN945B and p3-KP21315 (accession number CP124705.1) and pA1966-IMP (accession number MK036889.1) harboring *bla*_IMP-4_. Gray areas represent homologous parts between sequences. (**B**) Circular comparison of pKPN945B with other similar plasmids (pEr983-1, accession number CP060738.1); pKPN945B was used as a reference. (**C**) Circular comparison of pKPN945A with virulence plasmid of NTUH-K2044; NTUH-K2044 was used as a reference.

KPN945 carries a total of three plasmids, and we focused on the analysis of one of them, pKPN945B. pKPN945B is a 78,707-bp circular plasmid with 50.19% GC content, belonging to the IncR replicons and carrying multiple determinants of resistance (Table 2). We searched the NCBI database and found that pKPN945B shared 99.80% identity compared with the plasmid p3-KP21315 (IncR replicon) isolated from *K. pneumoniae* KP21315 but has only 68% query coverage. The remaining uncovered sequences were compared again to show high identity and coverage with pEr983-1 (IncFIB replicon, 99.99% identity, 96% coverage) and pA1966-IMP (IncF replicon, 100% identity, 100% coverage) (Fig. 2B). It can be inferred that *bla*_IMP-4_ was carried by a new IncR recombinant plasmid pKPN945B from isolate KPN945. The IncR backbone of pKPN945B is derived from plasmid p3-KP21315, and the drug-resistant region harboring *bla*_IMP-4_ is from pA1966-IMP, which belongs to IncF replicons.

The *bla*_IMP-4_ gene was located in a class one integron with the gene arrangement of IS*6100*-MobC-*ItrA-bla*_IMP-4_-*Intl1*-IS*26* (Fig. 2A). The genetic environments of the *bla*_IMP-4_ gene in pKPN945B and pA1966-IMP were very similar, with numerous genes associated with mobile genetic elements around *bla*_IMP-4_ of pKPN945B, such as *Intl1-Intl1*-IS*26*-IS*26*-integrase-recombinase. The presence of repeated IS*26* is a key factor in mediating the recombination of pA1966-IMP and p3-KP21315, with one originating from pA1966-IMP and the other from p3-KP21315 (Fig. 2A). We therefore speculate that IS*26* mediates different plasmid recombination, leading to the formation of a new resistant plasmid.

The pKPN945B belonging to the IncR replicon does not carry *tra* operons, but it can be transferred to other bacteria through conjugation. This differs from previous reports, which have shown that most IncR plasmids cannot be transferred into recipient bacteria via conjugation (18, 19, 33). Conjugation success may be due to other cofactors. We hypothesized that the non-conjugative plasmid pKPN945B can highjack the type IV secretion system of chromosomal elements (or ICEs) for transfer (34). Alternatively, MobC (plasmid mobilization relaxosome protein) could mediate plasmid mobilization (35). We also found the *oriT* region on pKPN945B (Fig. S4), and studies have shown that *oriT* sequences are essential for the mobilization of non-conjugative plasmids and that normally, non-mobilizable plasmids carrying *oriT* confer mobilizability on the plasmid (36). The discovery of the *oriT* sequence could reinforce the mobilization hypothesis of plasmid transfer. The *oriT* sequence of the plasmid was identified by oriTfinder (37). After alignment, *oriT* belonged to the *oriT*-R46 family (the ratio of matching length is 88%). We also predicted that the plasmid mobilization relaxosome protein MobC, whose interaction with *oriT*’s conserved nick region (nic), is critical in plasmid conjugation transfer (38). These suggest that pKPN945B is a potentially mobile plasmid. This hypothesis has yet to be further verified. Although pKPN945B, an IncR replicon, was transferred to EC600 through conjugation, the transconjugants KPN945J did not show MIC changes to most β-lactam antimicrobial, except for an increase in the MIC of IPM from 0.25 mg/L to 1 mg/L. The reason for the formation of this resistance pattern remains to be studied.

The modes of DNA replication include rolling circle replication and θ replication. Many plasmids replicate autonomously through a method called rolling circle replication. In this process, a replication initiator protein binds to a section of the double-stranded DNA called the origin of replication or *ori* and begins replication. In contrast, the replication mode of some bacterial DNA, such as *E. coli*, is θ replication. Current studies have shown that *rep*B is a rolling circle replication gene, while *rep*E is a θ replication gene (39). Most IncR replicons carry *rep*B (replication initiation protein), while pKPN945B carries *rep*E, both of which are responsible for plasmid replication. However, whether plasmid pKPN945B belongs to θ replication remains to be further verified. Moreover, most *rep*E are found in IncF replicons, which further indicated that pKPN945B may be a fusion plasmid. The pKPN945B also carries *resD* (involved in plasmid maintenance), *parAB* (involved in plasmid distribution and stability), *umuCD* (plays a role in SOS mutagenesis), and vagCD (encodes toxin-antitoxin systems involved in plasmid maintenance). This region is conserved in IncR plasmids and contributes to the stability of plasmid inheritance (14). The presence of MobC and putative *oriT* indicates that pKPN945B is mobilizable and may facilitate the spread of drug resistance genes.

Although we did not detect common virulence-related genes on the plasmid, we found that plasmid pKPN945A belonged to the IncHI1B/IncFIB(K) incompatibility group, with a total length of 225,147 bp and a GC content of 51.04%. IncHI1B/IncFIB(K) replicons were generally associated with pLVPK-like plasmids (40, 41), but no virulence factors such as *rmpA*/*A2*, *iucABCD*, and *iroBCD* were found in pKPN945A. pKPN945A and pLVPK plasmids of NTUH-K2044 showed 98.3% identity and 35% coverage through BLAST, so pKPN945A may partially evolve from pLVPK (Fig. 2C). pKPN945A carries copper resistance-related proteins (PcoS, PcoD, PcoB, and CopC), cation efflux system protein CusF (copper ion or silver ion), type II toxin-antitoxin system (VapC, HigB, and VapB), and RelB/StbD replicon stabilization protein (antitoxin to RelE/StbE). The bacterial toxin-antitoxin system plays an important role in bacterial multidrug tolerance and plasmid stability. Additionally, we found that pKPN945A carries the virulence factor *SrfB* (part of the surfactin antibiotic synthesis machinery) and ferric citrate uptake sigma factor regulator *FecR*, whose effects on the virulence of isolate KPN945 have yet to be verified. The genome of plasmid pKPN945A contains a large number of hypothetical proteins, insertion sequences, and transposons. The specific information about this plasmid needs to be further explored. Furthermore, KPN945 was highly resistant to serum killing and highly lethal to *G. mellonella*, showing a certain hypervirulence phenotype, but there are no classical virulence factors on the plasmid. The specific cause of the hypervirulence phenotype of isolate KPN945 remains to be further studied, and it also reminds us to be alert to the emergence of non-virulent plasmid-mediated hypervirulence *K. pneumoniae*.

### Phylogenetic analysis

**Fig 3:**
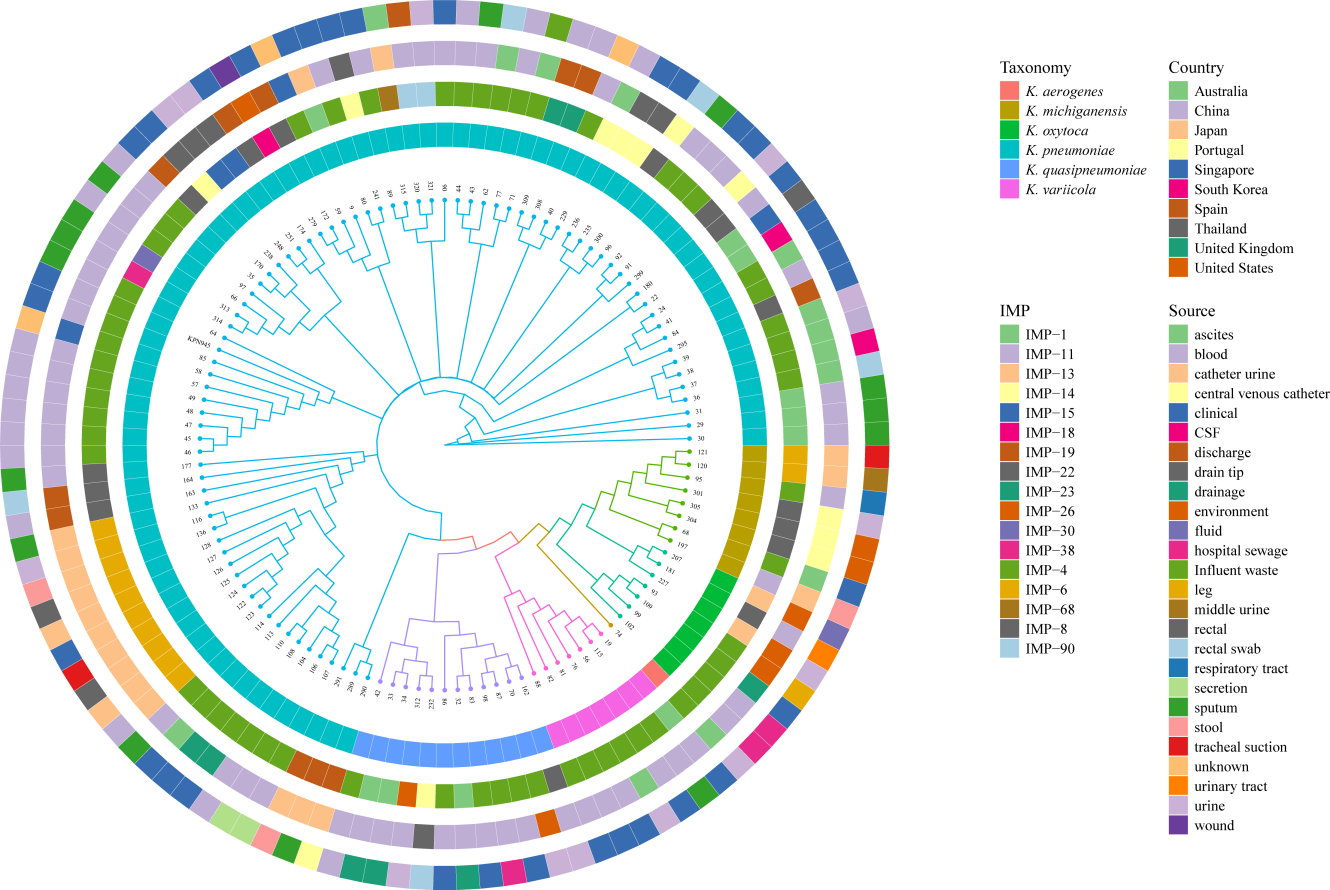
Phylogenetic tree of 118 isolates harboring *bla*_IMP_. Different colored branches on the tree represent different species of isolates. From the inner to outer circle represents the bacterial species, the specific IMP type, the country, and the isolate sources. The digital labels of the branches represent only the different strains. The specific information of the strains (IMP type, accession number, taxonomy, country, and source) is shown in Table S3.

We downloaded the whole genome sequences of 117 *bla*_IMP_-harboring *Klebsiella* spp. from the NCBI database and conducted a whole genome phylogenetic analysis, as shown in Fig. 3. Among the 118 *bla*_IMP_-harboring isolates, *K. pneumoniae* accounted for 70.4%, *K. aerogenes* for 0.8%, *Klebsiella michiganensis* for 6.8%, *Klebsiella oxytoca* for 5.9%, *K. quasipneumoniae* for 10.2%, and *Klebsiella variicola* for 5.9%. *K. pneumoniae* was the most common *bla*_IMP_ carrier among the *Klebsiella* spp. It is worth noting that *bla*_IMP-4_ accounted for the highest proportion (49.2%) among *Klebsiella* spp.; *bla*_IMP-6_ (10.2%), *bla*_IMP-1_ (8.5%), *bla*_IMP-8_ (7.6%), *bla*_IMP-14_ (5.1%), and *bla*_IMP-22_ (5.1%) also account for a certain proportion. There were also some less common IMP subtypes, such as *bla*_IMP-11_, *bla*_IMP-18_, *bla*_IMP-26_, and *bla*_IMP-30_. Among *K. pneumoniae*, *bla*_IMP-4_ was the most common subtype, presenting outbreaks around the world.

The prevalence of *bla*_IMP_-harboring *K. pneumoniae* was higher in Asian countries, mainly in China (43.4%), Japan (18.1%), Thailand, and Singapore. China was mainly endemic to *K. pneumoniae* harboring *bla*_IMP-4_. *K. pneumoniae* harboring *bla*_IMP-6_ and *bla*_IMP-14_ had the highest prevalence in Japan and Thailand, respectively. Consistent with previous reports (10), the countries with the highest prevalence of *bla*_IMP_ across all regions include China, Japan, Australia, the United States, Spain, Thailand, and Portugal. China and Japan account for the largest proportion (62.7%), with *bla*_IMP-4_ and *bla*_IMP-6_ being the most common types. *bla*_IMP_ is not only disseminated in clinical but also isolated in environmental samples such as sewage (Table S3). This reminds us to prevent cross-transmission between clinical and environmental settings. In particular, the high prevalence and isolation rates of *bla*_IMP-4_ deserve our attention.

### Conclusion

In conclusion, we report a hypervirulent CRKP carrying *bla*_IMP-4_, which is located on a novel transferable IncR plasmid. The IncR plasmid was formed by combining an IncR plasmid backbone with a drug-resistant region of an IncF plasmid via IS*26*. The high prevalence of *bla*_IMP-4_ and the emergence of novel transferable IncR plasmids harboring *bla*_IMP-4_ enrich our understanding of the broader genetic background of *bla*_IMP-4_ in China and suggest the need for improved clinical epidemiological surveillance to prevent its spread. Additionally, the emergence of hypervirulent CRKP mediated by non-virulent plasmids also deserves our attention.

## Data Availability

The data sets presented in this study can be found in online repositories. The names of the repository/repositories and accession number(s) can be found below: NCBI GenBank, CP142021-CP142024.

## SUPPLEMENTAL MATERIAL

Supplemental figures spectrum.00491-24-s0001.docx Fig. S1 to S4.

Table S1 spectrum.00491-24-s0002.docx Primers used in this study.

Table S2 spectrum.00491-24-s0003.docx Results of mCIM and eCIM of isolate KPN945.

Table S3 spectrum.00491-24-s0004.docx The specific information of the 118 isolates harboring blaIMP.

ASM does not own the copyrights to Supplemental Material that may be linked to, or accessed through, an article. The authors have granted ASM a non-exclusive, world-wide license to publish the Supplemental Material files. Please contact the corresponding author directly for reuse.
