# Supplementary material for: Characterization of pKPN945B, a novel transferable IncR plasmid from hypervirulent carbapenem-resistant Klebsiella pneumoniae, harboring blaIMP-4 and qnrS1
Source: Microbiol Spectr. 2024 Sep 17;12(11):e00491-24. doi: 10.1128/spectrum.00491-24 (PMC11537061; doi:10.1128/spectrum.00491-24)
Supplement: Supplemental figures — Fig. S1 to S4. [file spectrum.00491-24-s0001.docx]

**Supplementary Figures**

**
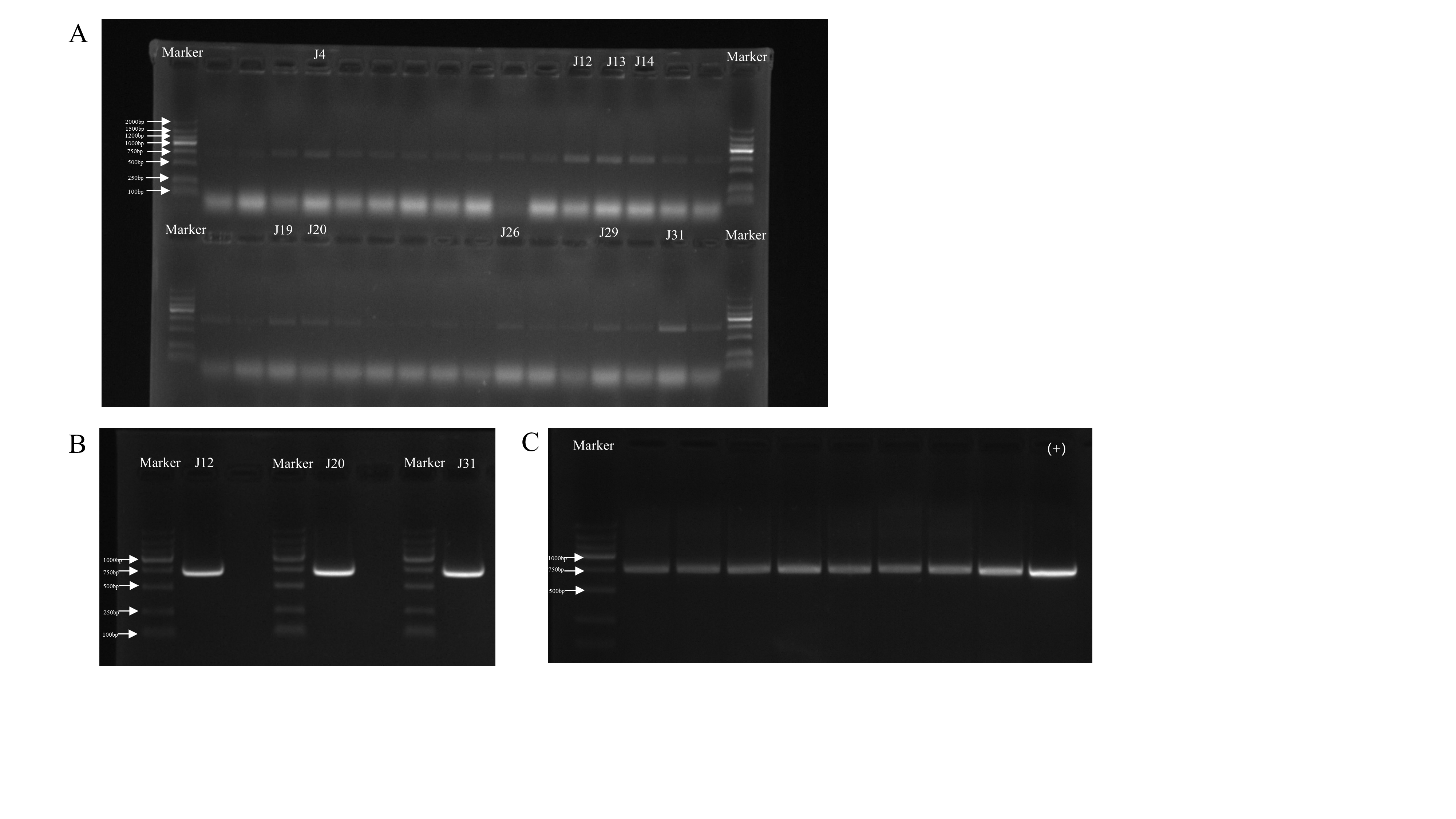
**

**Figure S1.** Conjugation result verification. (A) Conjugation screening results of EC600 as a recipient bacterium. Both ends of the lane are markers, and the middle lane is the colony PCR product of transconjugants (J4, J12, J13, J14, J19, J20, J26, J29, J31). Colonies growing on 500 mg/liter RIF +1 mg/liter MEM plate were randomly selected, identified by MALDI-TOF-MS, and colony PCR was used to determine whether *bla*_IMP-4_ (length: 741bp) was harbored. Agarose gel electrophoresis showed that the length of PCR products ranged from 500-750bp, indicating that the transconjugants might harbor *bla*_IMP-4_. Confirm the transconjugants harboring *bla*_IMP-4_ through Sanger sequencing, indicating successful conjugation. (B) The result of transconjugants' plasmid DNA. Three colony PCR-positive strains (J12, J20, J31) were selected to extract their plasmids, which were used as DNA templates for PCR, and agarose gel electrophoresis was performed to observe whether *bla*_IMP-4_ carried by pKPN945B was present in transconjugants' plasmids. (C) Conjugation screening results of ATCC13883 (RIF^R^) as a recipient bacterium. Lane 1 was marker, lane 2-9 was transconjugants (transconjugants' DNA was template and *bla*_IMP-4_ primer sequence was used for PCR), and lane 10 was positive control.

**
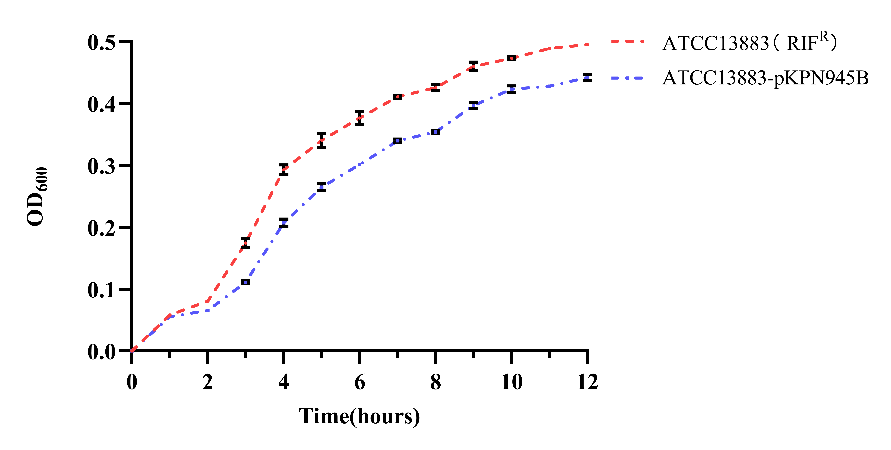
**

**Figure S2**. Growth kinetics curves of transconjugants ATCC13883-pKPN945B and recipient bacterium ATCC13883 (RIF^R^). There was significant difference in the growth curve between ATCC13883(RIF^R^) and ATCC13883-pKPN945B. A paired t-test was performed for each time point (*P* < 0.05).


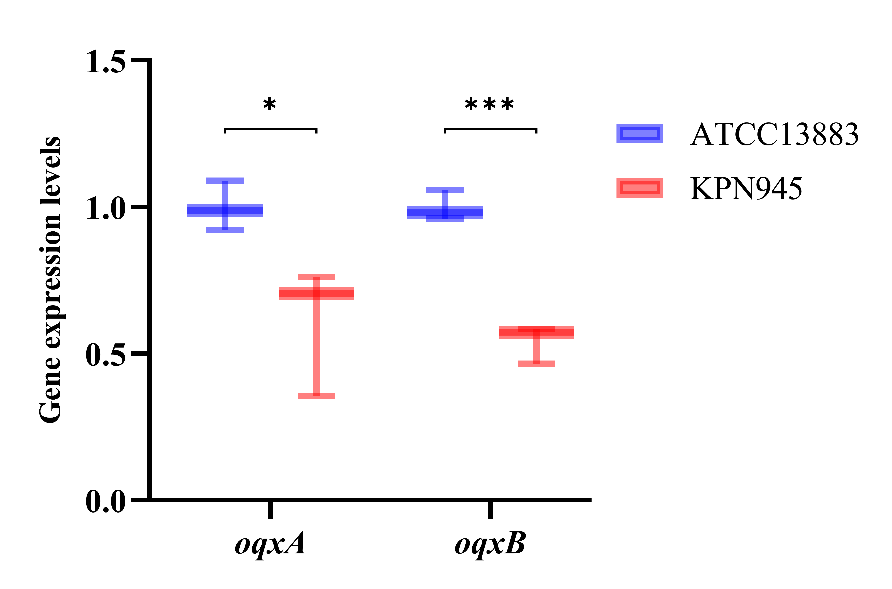


**Figure S3**. Relative expression level of efflux pump *oqxAB* of KPN945. * *P* < 0.05,

*** *P* < 0.001 by Multiple unpaired t tests.


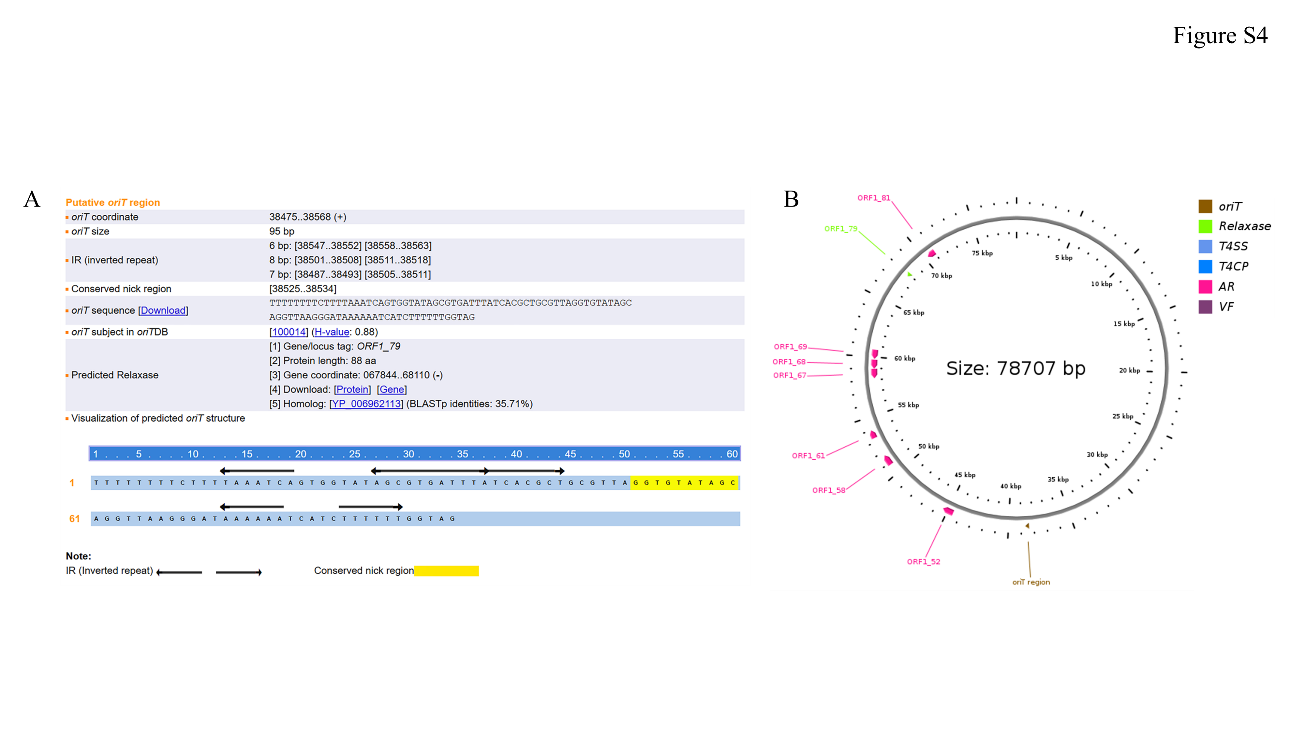


**Figure S4**. The result of *oriT* prediction. (A) List of the features of the putative *oriT* region: location, size, inverted repeat (IR), conserved nick region (nic), sequence, subject in *oriT*DB and predicted relaxase (after comparison on NCBI website, the sequence belonged to the plasmid mobilization relaxosome protein MobC). The detected nic site and IRs are displayed within the *oriT* sequence. The *oriT* was predicted by oriTfinder (<https://tool-mml.sjtu.edu.cn/oriTfinder/oriTfinder.html>). (B) The circular pKPN945B plasmid generated by the oriTfinder shows the locations and sizes of *oriT* (an origin of transfer region), the relaxase gene, and AR (antibiotic resistance) within this replicon. T4SS, type IV secretion system; T4CP, type IV coupling proteins; VF, virulence factors.
