# Supplementary material for: Characterization of pKPN945B, a novel transferable IncR plasmid from hypervirulent carbapenem-resistant Klebsiella pneumoniae, harboring blaIMP-4 and qnrS1
Source: Microbiol Spectr. 2024 Sep 17;12(11):e00491-24. doi: 10.1128/spectrum.00491-24 (PMC11537061; doi:10.1128/spectrum.00491-24)
Supplement: Table S1 — Primers used in this study. [file spectrum.00491-24-s0002.docx]

**Supplementary Table 1.** Primers used in this study.

| Application | Gene | Sequence | References |
| --- | --- | --- | --- |
| Carbapenemase genes | *bla*_KPC-2_ | F CATTCAAGGGCTTTCTTGCTGC  R ACGACGGCATAGTCATTTGC | (1) |
|  | *bla*_NDM_ | F CGGAATGGCTCATCACGATC  R GGTTTGGCGATCTGGTTTTC | (2) |
|  | *bla*_IMP_ | F TTAGTTGCTTAGTTTTGATGGTTTTTT  R ATGAGCAAGTTATCTGTATTCTTTATA | NA |
|  | *bla*_VIM_ | F TTATGGAGCAGCAACGATGT  R CAAAAGTCCCGCTCCAACGA | (3) |
|  | *bla*_OXA-48_ | F GCTTGATCGCCCTCGATT  R GATTTGCTCCGTGGCCGAAA | (4) |
| qRT-PCR | *oqxA* | F CAGGTGCTGTTCACGATAGATGAC  R CGGGAGACGAGGTTGGTATGG | NA |
|  | *oqxB* | F GTGGGTCTGGTGGTCCTGATG  R CCGCTTCCATGATGCCTTTCC | NA |
|  | *rpoB* | F AAGGATCTGCTCAGTGGTGTAATTC  R AAGACAACCTGTTCGTTCGTATCG | na |

Abbreviation: NA, not applicable.

**REFERENCE**

1. Dallenne C, Da Costa A, Decre D, Favier C, Arlet G. 2010. Development of a set of multiplex PCR assays for the detection of genes encoding important beta-lactamases in Enterobacteriaceae. J Antimicrob Chemother 65:490-5.<http://doi.org/10.1093/jac/dkp498>.

2. Krishnaraju M, Kamatchi C, Jha AK, Devasena N, Vennila R, Sumathi G, Vaidyanathan R. 2015. Complete sequencing of an IncX3 plasmid carrying blaNDM-5 allele reveals an early stage in the dissemination of the blaNDM gene. Indian J Med Microbiol 33:30-8.<http://doi.org/10.4103/0255-0857.148373>.

3. Kang J, Li G, Ma M, Lan M, Kang Y, Yang N, Jia W, Zhao Z. 2022. Evidence of Sharing of Carbapenem-Resistant Klebsiella pneumoniae Strains Between Intensive Care Unit Patients and the Environment. Infect Drug Resist 15:7831-7839.<http://doi.org/10.2147/IDR.S388085>.

4. Woodford N, Ellington MJ, Coelho JM, Turton JF, Ward ME, Brown S, Amyes SG, Livermore DM. 2006. Multiplex PCR for genes encoding prevalent OXA carbapenemases in Acinetobacter spp. Int J Antimicrob Agents 27:351-3.<http://doi.org/10.1016/j.ijantimicag.2006.01.004>.
