# Supplementary material for: Characterization of pKPN945B, a novel transferable IncR plasmid from hypervirulent carbapenem-resistant Klebsiella pneumoniae, harboring blaIMP-4 and qnrS1
Source: Microbiol Spectr. 2024 Sep 17;12(11):e00491-24. doi: 10.1128/spectrum.00491-24 (PMC11537061; doi:10.1128/spectrum.00491-24)
Supplement: Table S2 — Results of mCIM and eCIM of isolate KPN945. [file spectrum.00491-24-s0003.docx]

**Supplementary Table 2.** Results of mCIM and eCIM of isolate KPN945.

|  | MEM | MEM+EDTA |
| --- | --- | --- |
| KPN945 | 6 mm | 20 mm |

Abbreviation: mCIM, modified carbapenemase inactivation; eCIM, EDTA-modified carbapenemase inactivation.
