# Supplementary material for: Characterization of pKPN945B, a novel transferable IncR plasmid from hypervirulent carbapenem-resistant Klebsiella pneumoniae, harboring blaIMP-4 and qnrS1
Source: Microbiol Spectr. 2024 Sep 17;12(11):e00491-24. doi: 10.1128/spectrum.00491-24 (PMC11537061; doi:10.1128/spectrum.00491-24)
Supplement: Table S3 — The specific information of the 118 isolates harboring blaIMP. [file spectrum.00491-24-s0004.docx]

**Supplementary Table 3**. The specific information of the 118 isolates harboring *bla*_IMP_.

| number | IMP type | Accession number | Taxonomy | Country | Source |
| --- | --- | --- | --- | --- | --- |
| KPN945（this study) | IMP-4 | PRJNA1062447 | *Klebsiella pneumoniae* | China | clinical |
| 9 | IMP-1 | GCA_022267155.1 | *Klebsiella pneumoniae* | Japan | clinical |
| 19 | IMP-1 | GCA_030973025.1 | *Klebsiella variicola* | China | clinical |
| 22 | IMP-1 | GCA_031525115.1 | *Klebsiella pneumoniae* | Singapore | rectal |
| 24 | IMP-1 | GCA_032424395.1 | *Klebsiella pneumoniae* | South Korea | clinical |
| 29 | IMP-1 | GCA_030292325.1 | *Klebsiella pneumoniae* | China | sputum |
| 30 | IMP-1 | GCA_030292285.1 | *Klebsiella pneumoniae* | China | sputum |
| 31 | IMP-1 | GCA_030292245.1 | *Klebsiella pneumoniae* | China | sputum |
| 32 | IMP-1 | GCA_030292405.1 | *Klebsiella quasipneumoniae* | China | drainage |
| 33 | IMP-1 | GCA_030292365.1 | *Klebsiella quasipneumoniae* | China | drainage |
| 34 | IMP-1 | GCA_030292425.1 | *Klebsiella quasipneumoniae* | China | drainage |
| 45 | IMP-4 | GCA_000775955.1 | *Klebsiella pneumoniae* | China | blood |
| 46 | IMP-4 | GCA_001699025.1 | *Klebsiella pneumoniae* | China | blood |
| 47 | IMP-4 | GCA_001699045.1 | *Klebsiella pneumoniae* | China | blood |
| 48 | IMP-4 | GCA_001699095.1 | *Klebsiella pneumoniae* | China | blood |
| 49 | IMP-4 | GCA_001699105.1 | *Klebsiella pneumoniae* | China | blood |
| 36 | IMP-4 | GCA_002741685.1 | *Klebsiella pneumoniae* | Australia | rectal swab |
| 37 | IMP-4 | GCA_002753055.1 | *Klebsiella pneumoniae* | Australia | CSF |
| 38 | IMP-4 | GCA_002753075.1 | *Klebsiella pneumoniae* | Australia | blood |
| 39 | IMP-4 | GCA_002753555.1 | *Klebsiella pneumoniae* | Australia | urine |
| 40 | IMP-4 | GCA_002811335.4 | *Klebsiella pneumoniae* | China | NA |
| 35 | IMP-4 | GCA_008632115.1 | *Klebsiella pneumoniae* | China | blood |
| 41 | IMP-4 | GCA_008931345.1 | *Klebsiella pneumoniae* | Australia | clinical |
| 42 | IMP-4 | GCA_009498255.1 | *Klebsiella quasipneumoniae* | China | blood |
| 43 | IMP-4 | GCA_020496635.1 | *Klebsiella pneumoniae* | China | sputum |
| 44 | IMP-4 | GCA_020496675.1 | *Klebsiella pneumoniae* | China | blood |
| 56 | IMP-4 | GCA_022863695.1 | *Klebsiella variicola* | China | clinical |
| 57 | IMP-4 | GCA_023025805.1 | *Klebsiella pneumoniae* | China | blood |
| 58 | IMP-4 | GCA_023036595.1 | *Klebsiella pneumoniae* | Singapore | NA |
| 59 | IMP-4 | GCA_023042495.1 | *Klebsiella pneumoniae* | Singapore | NA |
| 62 | IMP-4 | GCA_023685635.1 | *Klebsiella pneumoniae* | Australia | rectal swab |
| 64 | IMP-4 | GCA_023978045.1 | *Klebsiella pneumoniae* | China | sputum |
| 66 | IMP-4 | GCA_024455135.1 | *Klebsiella pneumoniae* | China | blood |
| 68 | IMP-4 | GCA_025883475.1 | *Klebsiella michiganensis* | Australia | clinical |
| 70 | IMP-4 | GCA_029636165.2 | *Klebsiella quasipneumoniae* | USA | urine |
| 71 | IMP-4 | GCA_029813235.1 | *Klebsiella pneumoniae* | Australia | Influent waste… |
| 74 | IMP-4 | GCA_030183875.1 | *Klebsiella aerogenes* | Australia | urine |
| 76 | IMP-4 | GCA_030248925.1 | *Klebsiella variicola* | Australia | urine |
| 114 | IMP-4 | GCA_030292305.1 | *Klebsiella pneumoniae* | China | sputum |
| 115 | IMP-4 | GCA_030292385.1 | *Klebsiella variicola* | China | sputum |
| 77 | IMP-4 | GCA_030363395.1 | *Klebsiella pneumoniae* | China | blood |
| 80 | IMP-4 | GCA_030972215.1 | *Klebsiella pneumoniae* | China | clinical |
| 81 | IMP-4 | GCA_030972385.1 | *Klebsiella variicola* | China | clinical |
| 82 | IMP-4 | GCA_030972405.1 | *Klebsiella variicola* | China | clinical |
| 83 | IMP-4 | GCA_030972425.1 | *Klebsiella quasipneumoniae* | China | clinical |
| 84 | IMP-4 | GCA_030972435.1 | *Klebsiella pneumoniae* | China | clinical |
| 85 | IMP-4 | GCA_030973005.1 | *Klebsiella pneumoniae* | China | clinical |
| 86 | IMP-4 | GCA_030973085.1 | *Klebsiella quasipneumoniae* | China | clinical |
| 87 | IMP-4 | GCA_030973505.1 | *Klebsiella quasipneumoniae* | China | clinical |
| 88 | IMP-4 | GCA_030973525.1 | *Klebsiella variicola* | China | clinical |
| 89 | IMP-4 | GCA_031020955.1 | *Klebsiella pneumoniae* | China | clinical |
| 90 | IMP-4 | GCA_031317715.1 | *Klebsiella pneumoniae* | China | clinical |
| 91 | IMP-4 | GCA_031318855.1 | *Klebsiella pneumoniae* | China | clinical |
| 92 | IMP-4 | GCA_031318885.1 | *Klebsiella pneumoniae* | China | clinical |
| 93 | IMP-4 | GCA_031347465.1 | *Klebsiella oxytoca* | USA | leg |
| 95 | IMP-4 | GCA_031799765.1 | *Klebsiella michiganensis* | China | respiratory tract |
| 96 | IMP-4 | GCA_032460905.1 | *Klebsiella pneumoniae* | China | sputum |
| 97 | IMP-4 | GCA_032469835.1 | *Klebsiella pneumoniae* | China | sputum |
| 98 | IMP-4 | GCA_032740775.1 | *Klebsiella quasipneumoniae* | China | hospital sewage |
| 99 | IMP-4 | GCA_032745695.1 | *Klebsiella oxytoca* | China | hospital sewage |
| 102 | IMP-4 | GCA_032747375.1 | *Klebsiella oxytoca* | China | hospital sewage |
| 104 | IMP-4 | GCA_033119825.1 | *Klebsiella pneumoniae* | China | blood |
| 106 | IMP-4 | GCA_033434055.1 | *Klebsiella pneumoniae* | China | secretion |
| 107 | IMP-4 | GCA_033434075.1 | *Klebsiella pneumoniae* | China | secretion |
| 108 | IMP-4 | GCA_903935755.1 | *Klebsiella pneumoniae* | United Kingdom | clinical |
| 109 | IMP-4 | GCA_903935845.1 | *Klebsiella oxytoca* | United Kingdom | clinical |
| 110 | IMP-4 | GCA_903936195.1. | *Klebsiella pneumoniae* | United Kingdom | clinical |
| 113 | IMP-4 | GCF_008931565.1 | *Klebsiella pneumoniae* | Australia | clinical |
| 116 | IMP-6 | GCA_005405565.1 | *Klebsiella pneumoniae* | Japan | urine |
| 120 | IMP-6 | GCA_030359245.1 | *Klebsiella michiganensis* | Japan | middle urine |
| 121 | IMP-6 | GCA_030359285.1 | *Klebsiella michiganensis* | Japan | tracheal suction |
| 122 | IMP-6 | GCA_030359565.1 | *Klebsiella pneumoniae* | Japan | catheter urine |
| 123 | IMP-6 | GCA_030359605.1 | *Klebsiella pneumoniae* | Japan | blood |
| 124 | IMP-6 | GCA_030359645.1 | *Klebsiella pneumoniae* | Japan | drain tip |
| 125 | IMP-6 | GCA_030359665.1 | *Klebsiella pneumoniae* | Japan | tracheal suction |
| 126 | IMP-6 | GCA_030359725.1 | *Klebsiella pneumoniae* | Japan | clinical |
| 127 | IMP-6 | GCA_030359925.1 | *Klebsiella pneumoniae* | Japan | catheter urine |
| 128 | IMP-6 | GCA_030359955.1 | *Klebsiella pneumoniae* | Japan | drain tip |
| 133 | IMP-6 | GCA_030807425.1 | *Klebsiella pneumoniae* | Japan | sputum |
| 136 | IMP-6 | GCA_030808555.1 | *Klebsiella pneumoniae* | Japan | stool |
| 162 | IMP-8 | GCA_018604125.1 | *Klebsiella quasipneumoniae* | China | urine |
| 163 | IMP-8 | GCA_019218585.1 | *Klebsiella pneumoniae* | Spain | blood |
| 164 | IMP-8 | GCA_019218625.1 | *Klebsiella pneumoniae* | Spain | rectal swab |
| 170 | IMP-8 | GCA_024493275.1 | *Klebsiella pneumoniae* | Spain | clinical |
| 172 | IMP-8 | GCA_024887035.1 | *Klebsiella pneumoniae* | Spain | clinical |
| 174 | IMP-8 | GCA_024887375.1 | *Klebsiella pneumoniae* | Spain | clinical |
| 177 | IMP-8 | GCA_028581175.1 | *Klebsiella pneumoniae* | China | sputum |
| 180 | IMP-8 | GCA_030972705.1 | *Klebsiella pneumoniae* | China | clinical |
| 181 | IMP-8 | GCA_031799795.1 | *Klebsiella oxytoca* | China | urinary tract |
| 197 | IMP-11 | GCA_030800445.1 | *Klebsiella michiganensis* | Japan | stool |
| 207 | IMP-13 | GCA_017191755.3 | *Klebsiella oxytoca* | USA | fluid |
| 227 | IMP-13 | GCA_032457035.1 | *Klebsiella oxytoca* | USA | urine |
| 229 | IMP-14 | GCA_000817855.1 | *Klebsiella pneumoniae* | Australia | blood |
| 232 | IMP-14 | GCA_002246675.1 | *Klebsiella quasipneumoniae* | Thailand | rectal swab |
| 235 | IMP-14 | GCA_021981995.1 | *Klebsiella pneumoniae* | Thailand | clinical |
| 236 | IMP-14 | GCA_021983795.1 | *Klebsiella pneumoniae* | Thailand | clinical |
| 238 | IMP-14 | GCA_022268415.1 | *Klebsiella pneumoniae* | Thailand | clinical |
| 241 | IMP-14 | GCA_024943685.1 | *Klebsiella pneumoniae* | Thailand | clinical |
| 248 | IMP-15 | GCA_019927805.1 | *Klebsiella pneumoniae* | Thailand | urine |
| 251 | IMP-15 | GCA_023778315.1 | *Klebsiella pneumoniae* | Thailand | urine |
| 279 | IMP-18 | GCA_029621445.1 | *Klebsiella pneumoniae* | USA | wound |
| 289 | IMP-19 | GCA_029877445.1 | *Klebsiella pneumoniae* | Japan | sputum |
| 290 | IMP-19 | GCA_029877505.1 | *Klebsiella pneumoniae* | Japan | central venous catheter |
| 291 | IMP-19 | GCA_030796905.1 | *Klebsiella pneumoniae* | Japan | stool |
| 295 | IMP-22 | GCA_024886915.1 | *Klebsiella pneumoniae* | Spain | clinical |
| 299 | IMP-22 | GCA_032461135.1 | *Klebsiella pneumoniae* | Portugal | urine |
| 300 | IMP-22 | GCA_032462045.1 | *Klebsiella pneumoniae* | Portugal | rectal swab |
| 301 | IMP-22 | GCA_032676705.1 | *Klebsiella michiganensis* | Portugal | urine |
| 304 | IMP-22 | GCA_032676965.1 | *Klebsiella michiganensis* | Portugal | environment |
| 305 | IMP-22 | GCA_032677005.1 | *Klebsiella michiganensis* | Portugal | environment |
| 308 | IMP-23 | GCA_008867635.1 | *Klebsiella pneumoniae* | Spain | blood |
| 309 | IMP-23 | GCA_019218585.1 | *Klebsiella pneumoniae* | Spain | blood |
| 312 | IMP-26 | GCA_030292445.1 | *Klebsiella quasipneumoniae* | China | urine |
| 313 | IMP-30 | GCA_009755705.1 | *Klebsiella pneumoniae* | China | sputum |
| 314 | IMP-38 | GCA_009755705.1 | *Klebsiella pneumoniae* | China | sputum |
| 315 | IMP-68 | GCA_010367305.1 | *Klebsiella pneumoniae* | Japan | ascites |
| 320 | IMP-90 | GCA_030292265.1 | *Klebsiella pneumoniae* | China | discharge |
| 321 | IMP-90 | GCA_030292345.1 | *Klebsiella pneumoniae* | China | urine |
